# Novel Encapsulated Herbicide Delivery Mechanism: Its Efficacy in Mimosa Bush (*Vachellia farnesiana*) Control

**DOI:** 10.3390/plants10112505

**Published:** 2021-11-18

**Authors:** Amelia A. Limbongan, Shane D. Campbell, Victor J. Galea

**Affiliations:** School of Agriculture and Food Sciences, The University of Queensland, Gatton Campus, Gatton, QLD 4343, Australia; shane.campbell@uq.edu.au (S.D.C.); v.galea@uq.edu.au (V.J.G.)

**Keywords:** mimosa bush, control, chemical herbicides, encapsulation, implantation

## Abstract

Mimosa bush (*Vachellia farnesiana*) is an invasive woody weed widely distributed in Australia. While it can be controlled using several mechanical and chemical techniques, this study evaluated a novel herbicide delivery mechanism that minimizes the risk of spray drift and potential non-target damage. This method, developed by Bioherbicides Australia, involves the implantation of encapsulated granular herbicides into the stem of intact plants or into the stump after cutting off plants close to ground level (cut stumps). Trials were implemented near Moree (New South Wales, Australia) on intact (two experimental runs) plants and cut stumped (two experimental runs) plants. For each trial, an untreated control plus the conventional basal bark application of a liquid formulation of triclopyr + picloram mixed with diesel was included for comparison. Encapsulated glyphosate, aminopyralid + metsulfuron-methyl, hexazinone and clopyralid were also tested in all trials. In addition, encapsulated triclopyr + picloram, and metsulfuron-methyl were included in one of the intact plant trials. Aminopyralid + metsulfuron-methyl was consistently most effective on cut stump and intact plants, whilst clopyralid provided highest mortality when applied to cut stumps and single-stemmed intact plants. Particularly for multi-stemmed intact plants, clopyralid should be applied to each stem. Overall, the highest efficacy was achieved on single stemmed plants, but with further refinement of the technique, it should be possible to achieve similar results for multi-stemmed individuals. This method resulted in a reduction in the use of herbicide and environmental contamination while significantly improving speed of treatment.

## 1. Introduction

Mimosa bush (*Vachellia farnesiana* (L.) Wight & Arn.) is a naturalised species that has become widely distributed throughout northern Australia, particularly in grasslands and savannah areas [1]. At low population densities, it is generally not considered a problem for land managers and could have some benefits, such as providing an alternative animal feed during the dry season [2,3,4]. Nevertheless, mimosa bush has become a threat in areas where it forms large and dense infestations that compete with pasture grasses for moisture, soil nutrition and light. In high density populations, its thorny stems can also interfere with livestock access to water resources (e.g., dams) and disrupt the mustering of animals [5,6].

Despite the impacts of mimosa bush in the Australian context, there is limited published information on control options for this problematic weed. Currently some foliar [7,8,9], basal bark and cut stump applications using formulations of a limited range of herbicides are recommended for control of mimosa bush [10]. In Australia, only clopyralid is currently registered under a minor use permit (PER14929; expires September 2029) [11] for foliar applications. This approach is beneficial because of its speed of application, but it has potential for spray drift and off-target damage [12,13,14]. Basal bark spraying has proven effective for many woody weeds in Australia, including prickly acacia (*Vachellia nilotica*) [15], mesquite (*Prosopis* spp.) [16], parkinsonia (*Parkinsonia aculeata*) [17], calotrope (*Calotropis procera*) [18], white weeping broom (*Retama raetam)* [19] and mimosa bush [20]. For basal bark applications, herbicides (such as triclopyr + picloram and fluroxypyr) must be in an oil-soluble form allowing them to be mixed with diesel and sprayed around the full circumference of the base of the stem up to approximately 30 cm from the ground level [10]. The cut stump treatment is also an effective option for many woody weeds (including mimosa bush) and uses similar herbicides to those for basal bark applications. It has the advantage of being effective all year round but is time consuming and laborious [10,21,22].

Other techniques used for woody weeds include the ground application of residual granular (e.g., tebuthiuron) and liquid (hexazinone) herbicides. Dry formulations are applied using hand-operated scattering devices or power-driven spreaders, and for large areas aircraft application is an economic option [21,22]. The application is very simple and minimizes pesticide particle movement through the air. Unfortunately, residual effects tend to remain in the soil for a considerable period after application, which can result in soil pollution [23]. In Australia, there is currently a minor use permit (PER13891; expires March 2023) [24] allowing for the control of mimosa bush using tebuthiuron.

The stem injection technique targets the vascular bundle to transport the herbicide through the plant tissues. This approach is suitable for the thinning of native trees and the control of woody weeds. There are two traditional types of stem injection, which are the drill and fill method and the axe cut method (synonyms: hack-and-squirt; frill-and-spray). The drill and fill method uses a battery powered drill to make downward-angled holes into which a liquid herbicide formulation is placed, whereas the axe cut method uses an axe to make horizontal cuts to which the herbicide is surface applied [10]. A more recent innovation is the encapsulation of solid formulations of herbicides and their implantation into the stem of woody weeds using a specifically engineered device that drills, implants the capsule and plugs the hole. This technique is an alternative approach that has been designed to avoid un-necessary chemical exposure to the environment by ensuring placement and capture of the dose entirely within the target plant. Testing has been conducted on a range of species including prickly acacia (*V. nilotica*), leucaena (*Leucaena leucocephala*), *Eucalyptus saligna* and *E. dunnii* [25] while other species such as rubber vine (*Cryptostegia grandiflora*), athel pine (*Tamarix aphylla*) Chinese elm (*Celtis sinensis*), Chinee apple (*Ziziphus mauritiana*), Peruvian apple (*Cereus uruguayanus*) have also been successfully evaluated for compatibility with this method (unpublished data).

The control of mimosa bush is imperative due to the significant threat it poses to agricultural and grazing systems. To expand on the range of available control options, four trials were undertaken to evaluate the efficacy of chemical herbicide capsule application on mimosa bush when applied to the stem of intact plants or to the stump of plants after being cut off near ground level.

## 2. Materials and Methods

### 2.1. Site Details

**Figure 1:**
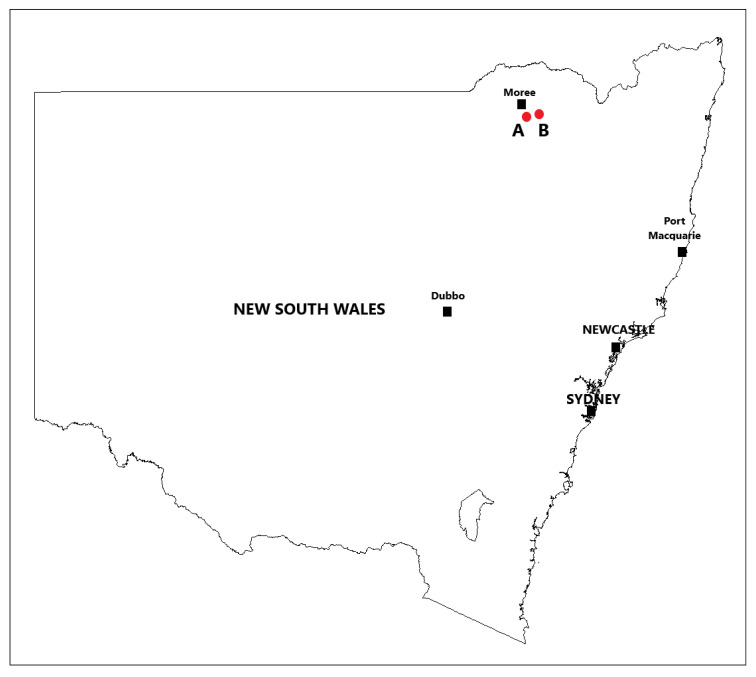
Intact plant and cut stump trial locations at Moree, NSW; (A) first round of trials (2018) and (B) second round of trials (2019).

The first intact plant and cut stump trials were conducted approximately 2 km (Figure 1) southeast of Moree, NSW, Australia (29°29′15″ S 149°53′13″ E). The site was located on a treeless plain that had a dense, uniform stand of small mimosa bush plants and an understory of native grass species. The land was designated a government stock route and is used as a transport corridor to move (walk or drove) livestock from one location to another as well as to serve as a public grazing resource in times of drought. As such, it was grazed only periodically. The second series of intact plant and cut stump trials were conducted approximately 27 km east of Moree, NSW (29°28′07″ S 150°04′53″ E). The site was in a Eucalypt woodland that had uniform, medium density stands of relatively large mimosa bush plants and an understory of native grass species.

The sites lie on the Upper Darling Plains which is surrounded by branching rivers notched into a regolith of alluvial sediments. The soils are dominated by Vertosols of moderate fertility. This type of soil has a good capability to transport and store water. Elevation of the area is 346 m AHD (Australian Height Datum) and the land is capable of supporting high impact land uses with intensive practical land management [26]. The region is dominantly covered by rain fed cropping and there are several parts of the area where shrubs such as mimosa bush have thickened [27].

### 2.2. Rainfall and Temperature

**Figure 2:**
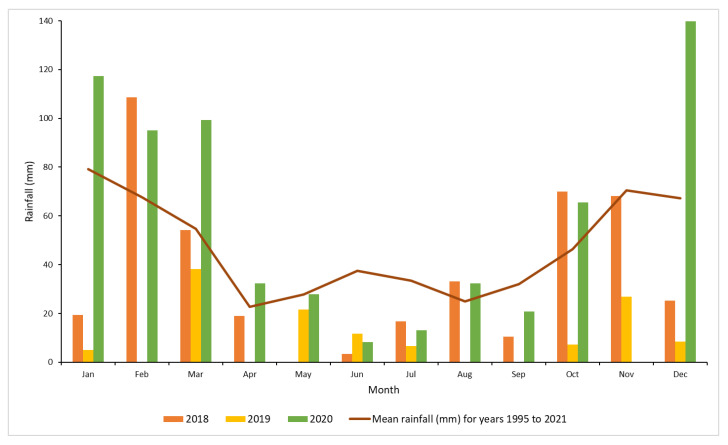
Monthly rainfall (mm) at Moree Airport for years 2018, 2019 and 2020 and the long term mean for Moree [28].

Monthly total rainfall data recorded at the Moree Airport weather station (the nearest to the experiment sites) were obtained from the Australian Bureau of Meteorology [28]. During the initial treatment period, the highest rainfall was obtained in October to December 2018, which is during the wet season. Throughout 2019, conditions were mostly dry, however high rainfall was recorded during the first three months of 2020 (Figure 2).

### 2.3. Intact Plant Trials

**Figure 3:**
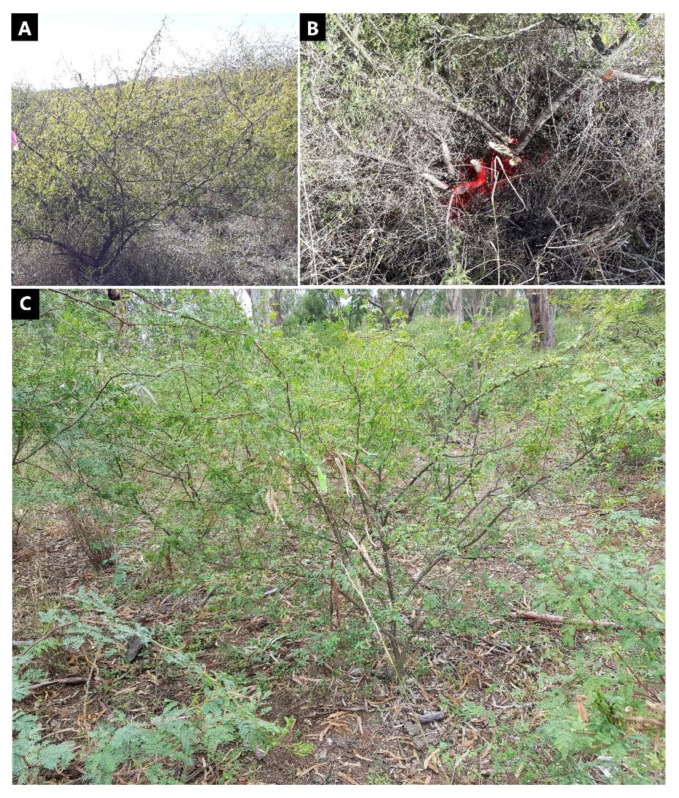
(**A**,**B**) Multi-stemmed plant in trial 1; (**C**) single-stemmed plant in trial 2.

The intact plant trials were established on 12 July 2018 and 29 March 2019, respectively, using a Randomized Complete Block Design. The 2018 trial (trial 1) incorporated eight herbicide treatments and four repetitions, whilst the 2019 trial (trial 2) had six herbicide treatments and four repetitions. Experimental units were groups of 15 mimosa bush plants that had their GPS location recorded and a plot placed on or close to the number first plant. The mimosa bush plants in trial 1 had an average height of 1.54 ± 0.04 (SE) m and canopy width of 2.19 ± 0.1 (SE) m. In trial 2, the plants were generally larger than those in trial 1, with an average height of 2.16 ± 0.11 (SE) m and canopy width of 3.09 ± 0.17 (SE) m. Furthermore, trial 1 plants were mostly multi-stemmed (Figure 3A,B), with an average number of 1.53 ± 0.07 (SE) stems, whereas in trial 2 the majority of plants were single-stemmed (Figure 3C).

The treatments comprised stem implantation of six (trial 1) or four (trial 2) encapsulated herbicides, a benchmark basal bark treatment of triclopyr + picloram mixed (Access^®^; Corteva Agriscience Pty Ltd., Sydney, NSW, Australia) with diesel, and an untreated control (Table 1).

For trial 2, the TyP treatment was not applied due to its poor efficacy in trial 1. Furthermore, treatments M (metsulfuron-methyl) and AM (aminopyralid + metsulfuron-methyl) demonstrated comparable mortality, therefore only one of these treatments (i.e., AM) was applied in subsequent trials.

**Figure 4:**
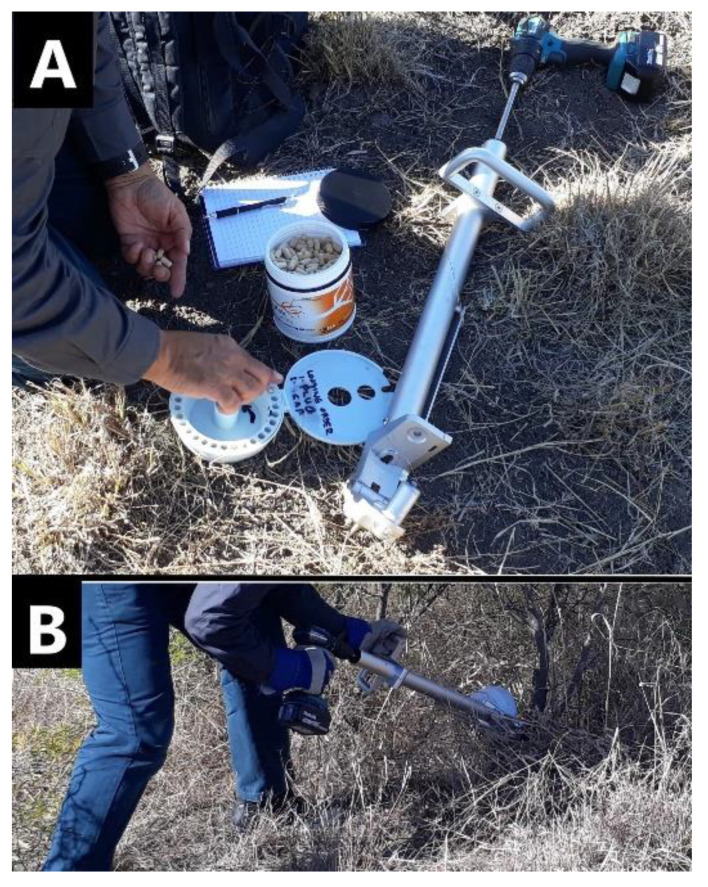
(**A**) Capsules are loaded into Injecta^®^ magazine followed by plugs; (**B**) stem implantation using Injecta^®^.

Herbicide capsules were manufactured by Bioherbicides Australia Pty Ltd [29] containing dry formulations of key herbicides typically used for control of woody weeds. The herbicide capsules were implanted using the Injecta^®^ (Bioherbicides Australia Pty Ltd, Brisbane, Queensland, Australia) capsule delivery method (Figure 4). The Injecta^®^ is a custom designed applicator with the following key components: the head unit with three sharp spikes to lock it firmly onto the plant surface; an 8 mm drill bit to bore a hole of 25 mm depth, a removable magazine which holds 30 herbicide capsules and sealing plugs, a body, handle, and shaft to which a cordless drill is attached. This device allows the operator to drill a hole and rapidly implant the capsule followed by the plug and the procedure takes between 5 and 8 s. The purpose of the plug is to seal the capsule into the plant which ensures that moisture remains in the wound area to avoid oxidation [25] and to keep the herbicide within the plant to prevent non-target damage. The sap from the plant reduces the integrity of the capsule and dissolves the herbicide. The herbicides were closely packed into size 0 hypromellose pharmaceutical-grade capsules (Suzhou Capsugel^®^; Suzhou Capsugel Co., Ltd., Suzhou, China). A hole is drilled into the plant, or for larger stems, at pre-determined intervals around the stem. To determine the number of capsules to apply to a plant, the circumference of the stems was measured near the base. One capsule was then applied to stems with a circumference up to 15 cm, two capsules were applied where the circumference was greater than 15 cm and less than 30 cm, and three capsules were applied where the circumference was greater than 30 cm and less than 45 cm. The capsules were then applied approximately 15-30 cm from ground level and details recorded on a plant-by-plant basis.

Basal bark application of herbicide mixture (Access^®^) was undertaken using a 5 L pressurized shoulder sprayer (Nylex^®^; Ames Australasia Pty Ltd., Melbourne, Victoria, Australia) with the nozzle adjusted to a coarse droplet spray. The whole (complete surface) of the lower 30 cm of every plant stem was sprayed to the point of runoff as per manufacturer instructions. The volume of herbicide applied was determined at the end of each treatment set and plants categorized by stem size class (by 15 cm increments) for all treatments. Time taken to apply treatments to each group of 15 plants was determined from GPS waypoint timestamps.

Monitoring of trial 1 was undertaken 3 months after treatment (MAT) on 18 October 2018, 8 MAT on 29 March 2019 and 15 MAT on 8 November 2019. For trial 2, monitoring was undertaken 8 MAT on 6 November 2019 and 20 MAT on 23 November 2020. Each time, an estimate of percentage mortality based on the whole canopy cover was undertaken. The canopy cover is formed by a group of individual plant aboveground parts, which include stem and leaves. If 100% mortality was recorded for two consecutive monitoring periods, the plant was classified as dead.

### 2.4. Cut Stump Trials

The cut stump trials (trials 3 and 4) were established on 13 July 2018 and 30 March 2019, respectively. For both trials, a Randomized Complete Block Design was used, but the number of repetitions varied. Trial 3 incorporated six herbicide treatments and three repetitions, while trial 4 had six herbicide treatments and four repetitions. Herbicide treatments for both trials were based on those used in the intact plant trials, and included four encapsulated herbicide treatments, a benchmark cut stump treatment of triclopyr + picloram mixed with diesel, and an untreated control (Table 2).

Experimental units were groups of 15 mimosa bush plants that had their GPS location recorded and a plot number placed on or close to the first plant. Mimosa bush plants in trial 3 had an average height of 1.54 ± 0.04 (SE) m and canopy width of 2.19 ± 0.1 (SE) m. The plants were mostly multi-stemmed, with an average of 1.53 ± 0.07 (SE) stems. In trial 4, the plants had an average height of 2.16 ± 0.11 (SE) m and canopy width of 3.09 ± 0.17 (SE) m. Most plants in this trial were larger and single-stemmed compared to those in trial 3.

Application of treatments involved cutting the stem of plants close to ground level (<15 cm above ground) using a pole pruning chainsaw (Ryobi^®^; Ryobi Ltd., Fuchu, Hiroshima, Japan). The stem was then marked with paint and numbered for ease of relocation during subsequent monitoring. The circumference of stems was also measured near the base to determine the appropriate herbicide dose, using the same approach as that described previously for the intact plant trials. Herbicide capsules were implanted and sealed into the cut stump ends. The benchmark triclopyr + picloram mixed with diesel was applied to the stump to the point of runoff. Number of stems and stem size class was recorded for all plants, along with herbicide volumes or capsules used.

For trial 3, monitoring was undertaken 4 MAT on 20 November 2018, 8 MAT on 29 March 2019 and 15 MAT on 8 November 2019. For trial 4, the assessments were completed 8 MAT on 7 November 2019 and 20 MAT on 24 November 2020. The main parameter for evaluation was stem regrowth and plant mortality.

### 2.5. Statistical Methods

The data was tested following General Linear Model (GLM) ANOVA using Minitab^®^ version 17. Treatment plot means were compared following Tukey’s test with 95% confidence. Arcsine transformation was applied to the canopy mortality to fulfill the statistical inference procedure in terms of normality of the data.

## 3. Results

### 3.1. Intact Plant Trials

**Figure 5:**
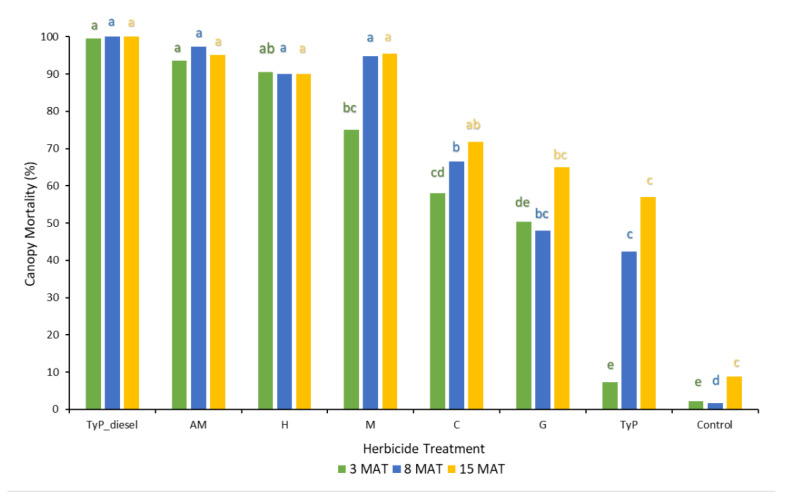
Mimosa bush canopy mortality 3, 8 and 15 MAT following herbicide applications to intact plants in trial 1. TyP_diesel, triclopyr + picloram mixed with diesel; AM, aminopyralid + metsulfu-ron-methyl; M, metsulfuron-methyl; H, hexazinone; C, clopyralid; G, glyphosate; TyP, triclopyr + picloram capsule Di-Bak TyP^TM^. For each monitoring time, columns with different letters indicate significant difference by Tukey’s test with 95% confidence.

In trial 1, significant treatment effects (*p* < 0.05) were recorded for all three monitoring times. Each stem received one capsule as all samples had a circumference less than 15 cm. Stem implantation of capsules containing aminopyralid + metsulfuron-methyl and hexazinone resulted in relatively high canopy mortality (≥90%) at 3 MAT, which continued across subsequent monitoring periods (Figure 5). Stem implantation using these encapsulated herbicides was equally as effective as the basal bark treatment (benchmark). Capsules containing only metsulfuron-methyl were not as effective as these treatments at 3 MAT, but they were at 8 MAT (94%) and thereafter. Clopyralid, glyphosate and triclopyr + picloram treatments all took time (15 MAT) to reach only a moderate level of canopy death, averaging 72%, 65 and 57%, respectively. Control plants remained relatively healthy despite the prolonged dry conditions with <9% canopy death recorded at 15 MAT (Figure 5).

Evaluation of herbicide use data based on equivalency in plant size (1 dose per 15 cm circumference) indicated that the application of capsules utilised 32% triclopyr and 22% of picloram (TyP capsules) compared to basal bark application of TyP (in diesel). Furthermore, application time using Injecta^®^ ranged between 30 and 50% that of basal bark spraying.

**Figure 6:**
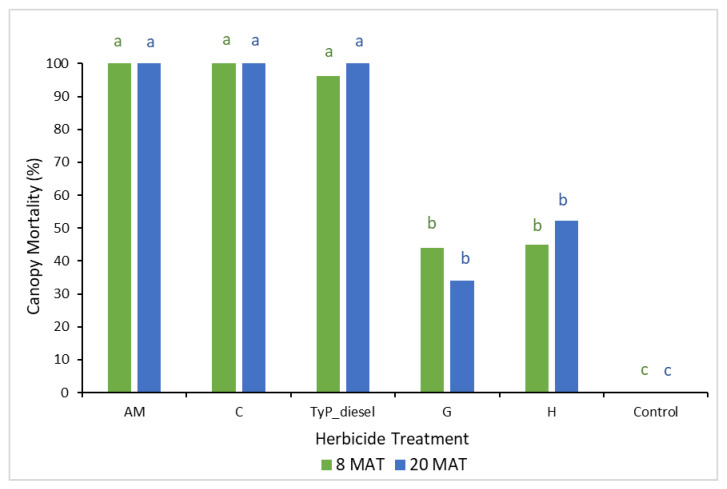
Mimosa bush canopy mortality 8 and 20 MAT following herbicide applications to intact plants in trial 2. AM, amino-pyralid + metsulfuron-methyl; C, clopyralid; TyP_diesel, triclopyr + picloram mixed with diesel; G, glyphosate; H, hexazinone. For each monitoring time, columns with different letters indicate significant difference by Tukey’s test with 95% confidence.

In trial 2, the mean number of capsules applied was 1.36 ± 0.03 (SE) per plant. Significant treatment effects (*p* < 0.05) were recorded for both the initial (8 MAT) and final assessments (20 MAT). Each time, stem implanted treatments of aminopyralid + metsulfuron-methyl and clopyralid resulted in the greatest canopy mortality (100%) (Figure 6). These treatments were as effective as basal bark application with triclopyr + picloram in diesel (benchmark). Herbicide treatments containing glyphosate and hexazinone took longer to cause maximum canopy mortality, but even at 20 MAT it was low, averaging only 34% and 52%, respectively. Control plants remained healthy throughout the trial.

### 3.2. Cut Stump Trials

**Figure 7:**
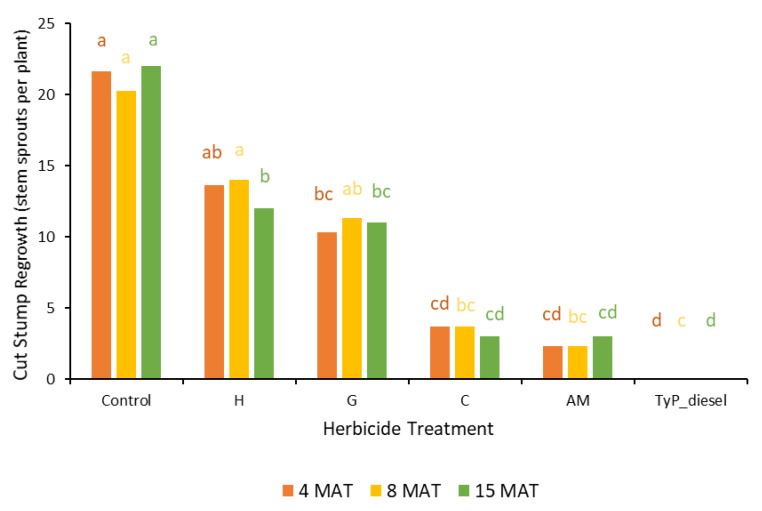
Cut stump regrowth (stem sprouts per plant) of mimosa bush 3, 8 and 15 MAT following cut stump herbicide application in trial 3. H, hexazinone; G, glyphosate; C, clopyralid; AM, aminopyralid + metsulfuron-methyl; TyP_diesel, triclopyr + picloram mixed with diesel. For each monitoring time, columns with different letters indicate significant difference by Tukey’s test with 95% confidence.

**Figure 8:**
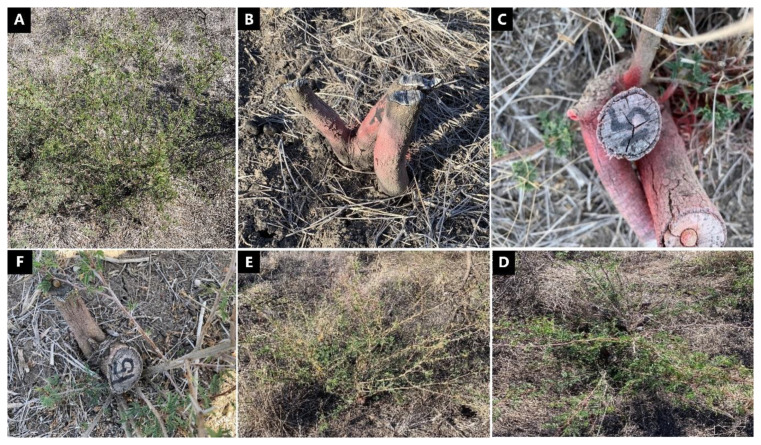
Plant response 15 MAT following cut stump applications in trial 3, clockwise: (**A**) Control (untreated sample), (**B**) triclopyr + picloram mixed with diesel treatment, (**C**) aminopyralid + metsulfuron-methyl treatment, (**D**) hexazinone treatment, (**E**) glyphosate treatment and (**F**) clopyralid treatment. Photo credit: Dr. Shane Campbell.

In trial 3, significant treatment effects (*p* < 0.05) were recorded for all three monitoring times (Figure 7). Similar to trial 1, each stump received one capsule as all samples had a circumference less than 15 cm. The traditional cut stump application using triclopyr + picloram with diesel gave significantly greater control of mimosa bush, with no regrowth recorded at any monitoring period. Treatment with encapsulated herbicides was most effective using clopyralid and aminopyralid + metsulfuron-methyl, with stem regrowth across the three monitoring periods ranging between two and four stems. Hexazinone and glyphosate were least effective with plants having an average of more than 11 stems at 15 MAT (Figure 7 and Figure 8).

**Figure 9:**
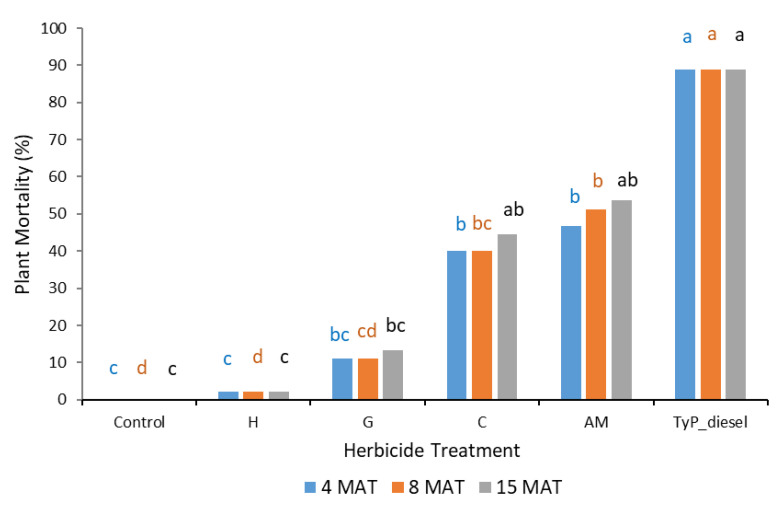
Plant mortality of mimosa bush 3, 8 and 15 MAT following cut stump herbicide application in trial 3. H, hexazinone; G, glyphosate; C, clopyralid; AM, aminopyralid + metsulfuron-methyl; TyP_diesel, triclopyr + picloram mixed with diesel. For each monitoring time, columns with different letters indicate significant difference by Tukey’s test with 95% confidence.

Plant mortality displayed a similar trend to that of stem regrowth, with clopyralid and aminopyralid + metsulfuron-methyl giving the best results, although only moderate mortality (44% to 54%) was recorded at 15 MAT (Figure 9). Hexazinone and glyphosate exhibited even lower efficacy, with plant mortality at 15 MAT averaging only 2% and 13%, respectively.

**Figure 10:**
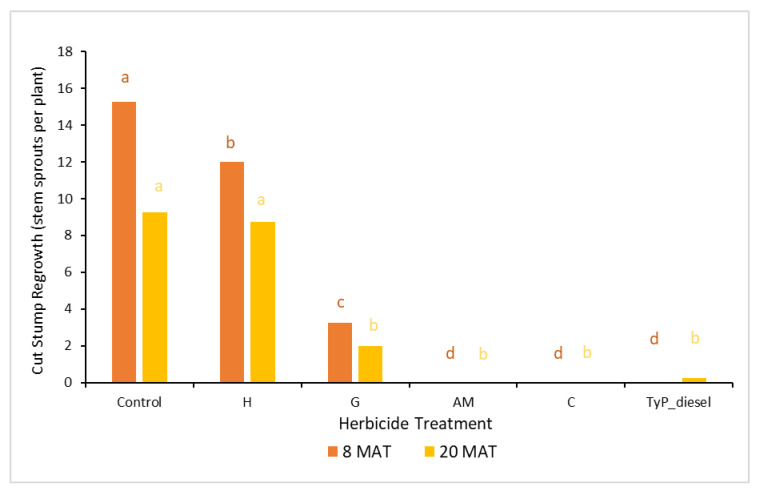
Average stem regrowth of mimosa bush 8 and 20 MAT following cut stump herbicide application in trial 4. H, hexazinone; G, glyphosate; AM, aminopyralid + metsulfuron-methyl; C, clopyralid; TyP_diesel, triclopyr + picloram mixed with diesel. For each monitoring time, columns with different letters indicate significant difference by Tukey’s test with 95% confidence.

In trial 4, significant treatment effects (*p* < 0.05) were also recorded for the initial assessment at 8 MAT. The number of capsules applied was 1.57 ± 0.04 (SE) per stem. As for trial 3, stem implantation treatments of aminopyralid + metsulfuron-methyl and clopyralid resulted in the least stem regrowth, which was not significantly different to the traditional cut stump treatment using triclopyr + picloram with diesel (benchmark). All three treatments had no new stem regrowth from the cut stump at 8 MAT. By contrast, hexazinone was the least effective treatment with plants having an average of 12 stems (Figure 10).

The final assessment at 20 MAT displayed similar results to the 8 MAT for most treatments, with stem implantation of aminopyralid + metsulfuron-methyl and clopyralid remaining not significantly different (*p* > 0.05) to the benchmark treatment. A slight, but non-significant reduction occurred with the glyphosate treatment which had significantly more stems than the benchmark treatment 8 MAT but not 20 MAT (Figure 10).

**Figure 11:**
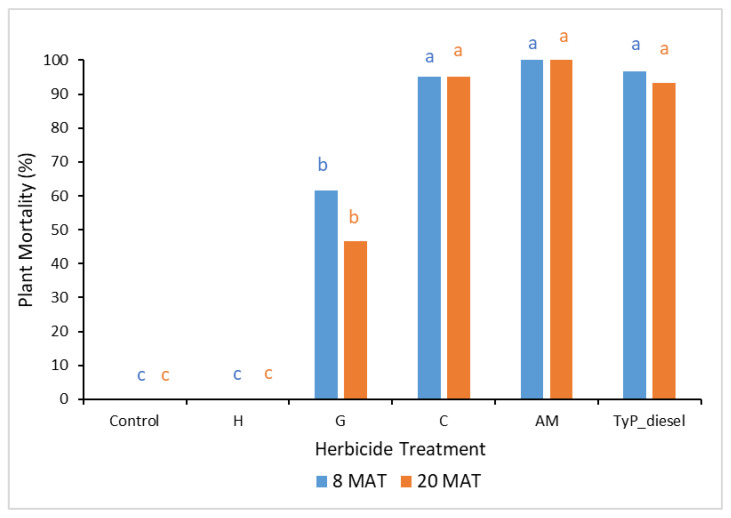
Plant mortality of mimosa bush 8 and 20 MAT following cut stump herbicide application in trial 4. H, hexazinone; G, glyphosate; C, clopyralid; AM, aminopyralid + metsulfuron-methyl; TyP_diesel, triclopyr + picloram mixed with diesel. For each monitoring time, columns with different letters indicate significant difference by Tukey’s test with 95% confidence.

In considering mortality (Figure 11), plants treated with clopyralid and aminopyralid + metsulfuron-methyl as well as the benchmark displayed the highest mortality at 20 MAT, averaging 95%, 100% and 93%, respectively. Glyphosate displayed moderate mortality (47%), whilst hexazinone failed to kill any mimosa bush.

## 4. Discussion

The results suggest that the application of encapsulated dry formulation herbicides using the Injecta^®^ capsule delivery technique can provide control of mimosa bush that is comparable to basal bark and cut stump treatments using triclopyr + picloram mixed with diesel. The plants were able to dissolve the capsules and absorb the granulated herbicide, whilst the inserted plugs sealed the chemical inside the stem and minimized the risk of off target damage. For the operator, this technique also prevented direct exposure to the herbicide and required minimal personal protective equipment (PPE) compared to other techniques [25,30,31].

Clopyralid, tebuthiuron, fluroxypyr and triclopyr + picloram are the currently recommended herbicides for mimosa bush control in Australia [10,32]. Of these, triclopyr + picloram was selected as the benchmark treatment to compare the encapsulated dry formulation herbicides against basal bark. It is a very popular herbicide for cut stump and basal bark applications of woody weeds, such as mimosa bush. Tebuthiuron was not considered for use in these trials because of its potential to harm non-target plants and risks to local ecology [33,34].

As expected, minimal mortality (<10%) occurred if mimosa bush plants received no herbicide treatments, even if they were cut-off close to ground level. A similar response has been recorded for several woody weeds, such as Chinese privet (*Ligustrum sinense* Lour.) (14% mortality) [35] and tree of heaven (*Ailanthus altissima* (Mill.) Swingle) (21% mortality) [36], if herbicide was not applied to the cut stump. In contrast, the benchmark basal bark treatment of triclopyr + picloram (Access^®^) mixed with diesel provided excellent control (>89%). Diesel is a very efficient carrier which simplifies the application of the liquid herbicide into the target plant and reduces evaporation of spray droplets after they leave the sprayer [37,38,39]. Compared to the liquid formulation of triclopyr + picloram mixed with diesel, the encapsulated formulation of triclopyr + picloram was ineffective for mimosa bush control when implanted into the stem of plants. This was surprising, as liquid herbicides containing triclopyr + picloram have been widely recommended and used for stem injection of a range of exotic and native woody plants in Australia [40].

Glyphosate is another herbicide that is commonly used for stem injection applications to control woody weeds and implantation of encapsulated glyphosate has given promising results on several species including prickly acacia (*V. nilotica*), another weed belonging to the same genus as mimosa bush [41]. However, in both the intact and cut stump trials, glyphosate gave only poor to moderate control and does not warrant further testing on mimosa bush, given that there are much more effective alternatives. This finding illustrates the importance of specific studies to ensure that only the most effective herbicides progress to the registration stage for a particular weed and application technique.

Overall, aminopyralid + metsulfuron-methyl was the most effective of the encapsulated herbicides for mimosa bush control across all intact and cut stump trials, followed by clopyralid. Currently, clopyralid is only recommended for foliar application on actively growing mimosa bush plants in full leaf [32], whilst aminopyralid + metsulfuron is also generally considered a foliar herbicide. However, as mentioned previously encapsulated formulations of aminopyralid + metsulfuron are proving to be highly effective when implanted into a range of woody weeds (unpublished data), including mimosa bush. Metsulfuron-methyl and hexazinone were two other herbicides that individually performed well in at least one of the trials. We did not continue to include metsulfuron-methyl on its own beyond trial 1 given the high concentration present in aminopyralid + metsulfuron-methyl. However, whilst it tended to act slower than when present with aminopyralid, it eventually caused similar mortality levels. Consequently, future studies on mimosa bush should include herbicides containing metsulfuron-methyl on its own as well as in combination with aminopyralid, to better understand whether inclusion of aminopyralid is necessary or not.

Hexazinone was slower acting than the other herbicides in the intact plant experiments (trial 1 and 2), until sufficient rainfall occurred between October to December 2018 to fully activate it. In trial 1, hexazinone showed similar results with the benchmark. However, in trial 2, hexazinone displayed the least canopy mortality compared to the other treatments. The plants treated with hexazinone might have recovered from hexazinone treatment effect as low rainfall occurred a few months before the assessment at 20 MAT. For the cut stump method, the efficacy of hexazinone was minimal in both trials (trial 3 and 4) and not significantly different to the untreated control. With removal of the canopy of the mimosa bush plants during the cut stump process, photosynthesis did not occur in the plant which lead to low efficacy of hexazinone [42,43]. Cut stumped plants managed to sustain life by concentrating on cell division and elongation, and stimulated more stem regrowth from the vegetative buds [44].

Overall, the herbicide applications tended to be less effective in the first round of trials (trial 1 and 3) compared to the second-round trials (trial 2 and 4). The plants in first round trials (trial 1 and 3) were generally small in size (circumference less than 15 cm) but multi-stemmed, which appeared to reduce herbicide efficacy. Given the small size of the plants and the method used to determine the number of capsules to apply in each case (see Section 2.3), not all stems were implanted with a capsule. Consequently, the herbicide affected the treated stems, but it did not always translate into effective control of the whole (multi-stemmed) plant. In contrast, plants in trial 2 and trial 4 were mostly single-stemmed, which resulted in greater compatibility between the circumference of the stem and the dose applied [25]. Similar results occurred for the control of common ragweed (*Ambrosia artemisiifolia*) using a combination of clopyralid application with cutting treatments [45]. An experiment of sweetbrier (*Rosa rubiginosa*) control was also not effective on multi-stemmed plants [38,46]. The control of tree of heaven (*A. altissima)* using cut stump and basal bark herbicide applications and the control of Chinese privet (*L. sinense*) also revealed that plant circumference affected herbicide efficacy [35,47]. However, there are instances where only treating a single stem of a multi-stemmed plant has been successful. For example, multi-stemmed plants of European buckthorn (*Rhamnus cathartica*) were effectively controlled by applying glyphosate, imazapyr or picloram to a single stem that had been either cut off or girdled [48]. In the case of mimosa bush, it appears that it may be necessary to treat all stems on multi-stemmed plants to achieve maximum efficacy, which warrants further investigation.

The findings of this study suggest that this technique is worth progressing further as an effective control option for mimosa bush, using those herbicides that demonstrated high efficacy across the four trials. While efficacy was greatest on infestations containing predominately single stemmed plants or those with only a few stems, the exploration of increasing dosage or reviewing dose placement for multi-stemmed plants should increase efficacy. Once refinements to the technique have been made, cost and benefit comparisons with other techniques would be recommended to assist land managers to decide on the most cost-effective techniques for their situation.

## 5. Conclusions

This study demonstrated the ability to control mimosa bush through implantation of encapsulated herbicides into either the stem of intact plants or into cut stumps, and for reducing the amount of herbicide required to kill this species. Several herbicides proved capable of causing high mortality of mimosa bush in at least one of the four trials undertaken (aminopyralid + metsulfuron-methyl, hexazinone, metsulfuron-methyl and clopyralid). However, aminopyralid + metsulfuron-methyl consistently gave the highest mortality across all intact plant and cut stump trials, achieving comparable results to basal bark or cut stump applications using triclopyr + picloram mixed with diesel. Overall, highest efficacy was achieved on single stemmed plants, but with some further refinement of the technique it should be possible to achieve similar results for multi-stemmed species.

Further research is now needed to determine the situations where this technique would be a cost-effective option for control of mimosa bush compared to other available options. Key factors to consider include infestation (size and density) and plant characteristics (size and number of stems), but in some situations the usefulness of this technique for minimising spray drift and non-target damage may also be an important consideration.

## Figures and Tables

**Table 1:** Chemical herbicide treatments for the intact plant trials.

Treatment	Description	Dose of Product per Capsule	Active Ingredient Concentration in Product
Control	Untreated plants	No treatment	
TyP_diesel	Conventional/basal bark application of triclopyr + picloram mixed with diesel	Diluted 1:60	Triclopyr 240 g/L Picloram 120 g/L
G	Stem implanted with Di-Bak G™	350 mg glyphosate/capsule	700 g/kg
AM	Stem implanted with Di-Bak AM™	155 mg aminopyralid + 125 mg metsulfuron-methyl/capsule	375 g/kg 300 g/kg
H	Stem implanted with Di-Bak H™	350 mg hexazinone/capsule	750 g/kg
TyP	Stem implanted with Di-Bak TyP™	120 mg triclopyr + 40 mg picloram/capsule	300 g/kg 100 g/kg
C	Stem implanted with Di-Bak C™	450 mg clopyralid/capsule	750 g/kg
M	Stem implanted with Di-Bak M™	330 mg metsulfuron-methyl/capsule	600g/kg

**Table 2:** Chemical herbicide treatments for cut stump trials.

Treatment	Description	Dose of Product per Capsule	Active Ingredient Concentration in Product
Control	Cut stumps without implanted herbicide capsule	No chemical herbicide treatment	
TyP_diesel	Cut stumps sprayed with triclopyr + picloram mixed with diesel	Diluted 1:60	Triclopyr 240 g/L Picloram 120 g/L
G	Cut stumps implanted with Di-Bak G™	350 mg glyphosate/capsule	700 g/kg
AM	Cut stumps implanted with Di-Bak AM™	155 mg aminopyralid + 125 mg metsulfuron-methyl/capsule	375 g/kg 300 g/kg
H	Cut stumps implanted with Di-Bak H™	350 mg hexazinone/capsule	750 g/kg
C	Cut stumps implanted with Di-Bak C™	450 mg clopyralid/capsule	750 g/kg

## Data Availability

Not applicable.
